# The Regulatory Role of IL-10 in Neurodegenerative Diseases

**DOI:** 10.3390/biom10071017

**Published:** 2020-07-09

**Authors:** Chiara Porro, Antonia Cianciulli, Maria Antonietta Panaro

**Affiliations:** 1 Department of Clinical and Experimental Medicine, University of Foggia, 71122 Foggia, Italy; chiara.porro@unifg.it; 2 Department of Biosciences, Biotechnologies and Biopharmaceutics, University of Bari, 70125 Bari, Italy; antonia.cianciulli@uniba.it

**Keywords:** IL-10, neurodegeneration, neuroinflammation, microglia, signaling

## Abstract

IL-10, an immunosuppressive cytokine, is considered an important anti-inflammatory modulator of glial activation, preventing inflammation-mediated neuronal degeneration under pathological conditions. In this narrative review, we summarize recent insights about the role of IL-10 in the neurodegeneration associated with neuroinflammation, in diseases such as Multiple Sclerosis, Traumatic Brain Injury, Amyotrophic lateral sclerosis, Alzheimer’s Disease, and Parkinson’s Disease, focusing on the contribution of this cytokine not only in terms of protective action, but also as possibly responsible for clinical worsening. The knowledge of this double face of the same coin, regarding the biological role of the IL-10, could aid the development of targeted therapies useful for limiting neurodegenerative processes.

## 1. Introduction

Interleukin (IL)-10, a potent anti-inflammatory cytokine, plays a critical role in balancing immune responses in order to circumvent chronic inflammatory diseases [1]. It was first described after the analysis for secreted factors by immunomodulatory CD4+ T helper 2 (Th2) mouse lymphocytes (named as anti-inflammatory cells) that can regulate CD4+ T helper 1 (Th1) lymphocytes (reported as pro-inflammatory cells) [2,3].

IL-10 acts in innate as well as in adaptive immunity, both in terms of immunosuppressive and immunostimulatory effects, thus, regulating response in many cell types, such as antigen-presenting cells (APCs), including dendritic cells (DCs), Langerhans cells, and macrophages [4].

Inflammatory responses play a central role in the pathophysiology of several neurodegenerative diseases. In this regard, neuroinflammation is characterized by the activation of resident glial cells, committed to central nervous system (CNS) immune surveillance, through the release of cytokines, chemokines, and other mediators, which, in turn, are able to recruit peripheral cells, including lymphocytes, monocytes and neutrophils [5,6].

In the course of CNS pathology, the levels of IL-10 significantly increase in the brain in order to ensure nervous tissue survival and mitigate inflammatory responses triggering several signaling pleiotropic pathways [7].

Here, we present a narrative review on the role of IL-10 in promoting the resolution of inflammatory cascades that are important for the brain integrity, focusing on the possible usefulness of IL-10 as a therapeutic or potential biomarker during neurodegenerative diseases.

## 2. Biological Activity of IL-10

IL-10, also known as human cytokine synthesis inhibitory factor (CSIF), is a homodimeric polypeptide of 17 kDa and is encoded in humans by the *IL10 gene*, located on chromosome 1 and comprising 5 exon [8]. IL-10 is a type II cytokine in a family that includes: IL-19, IL-20, IL-22, IL-26, and IL-29. All these cytokines exhibit similar gene organization as well as bind to receptors of similar structure, although with different biological activities [8].

IL-10 has been described to be produced by almost all leukocytes, including all T cell subsets, monocytes, macrophages, neutrophils, eosinophils, mast cells, dendritic cells (DCs), B and natural killer (NK) cells [9]. In addition, keratinocytes and epithelial cells are reported to release IL-10 in response to infectious insult, tissue damage and the presence of tumor cells [10].

This wide source of IL-10 production clearly underscores its physiologic significance, so many cell types able to produce IL-10 could ensure its rapid availability as well as the complexity of its regulation and could explain its important modulatory activity in a plethora of pathophysiological conditions.

IL-10 is generally reported as an anti-inflammatory cytokine with multiple immunoregulatory effects particularly important during the resolution phase [11]. In fact, IL-10 is able to inhibit the production of several inflammatory cytokines, such as TNF-α, IL-1β, IL-6 and IFN-γ secretion from Toll-Like Receptor (TLR)-triggered myeloid lineage cells [12,13,14]. Moreover, apart from dampening the expression of Th1 cytokines, IL-10 is a powerful inhibitor of antigen presentation, since it can reduce the expression of the major histocompatibility complex class II (MHC II) and the co-stimulatory molecules CD80 on macrophages and CD86 on dendritic cells’ surfaces. In addition, IL-10 enhances B cell proliferation and antibody production [13], and can inhibit the reactive oxygen species (ROS) generation other than increasing the release of TNF receptors, which may antagonize the effects of TNF-α [12].

## 3. Signal Transduction Pathway of IL-10

When IL-10 is produced and secreted, it acts specifically on the functional receptor complex of IL-10, which is composed of two subunits, IL-10R1 (IL-10Rα) and IL-10R2 (IL-10Rβ), both members of the interferon receptor (IFNR) family [15]. IL-10Rα is a cell surface receptor with a single transmembrane domain and binds IL-10 with high affinity (Kd ~35–200 pM). The IL-10Rα chain is expressed by most hemopoietic cells and at high levels on both macrophages and DCs. Interestingly, IL-10R1 expression has been also described in non-hemopoietic cells, although it is expressed in an inducible rather than constitutive form. In this regard, IL-10R1 expression was detected in LPS-induced fibroblasts, as well as in epidermal cells or keratinocytes after treatment with glucocorticoids or dihydroxy-vitamin D3 [14].

The second subunit of the IL-10R complex, IL-10Rβ, was identified and characterized by Kotenko et al. [16] and later, it was observed in a model murine that a disruption of the IL-10Rβ chain led to IL-10 unresponsiveness [17].

The IL-10Rβ chain is expressed in a ubiquitous and constitutive manner by all cell types [14] and its principal function seems to be the recruitment of a Janus (Jak) kinase/tyrosine kinase (Tyk) for the signaling pathway [16].

**Figure 1:**
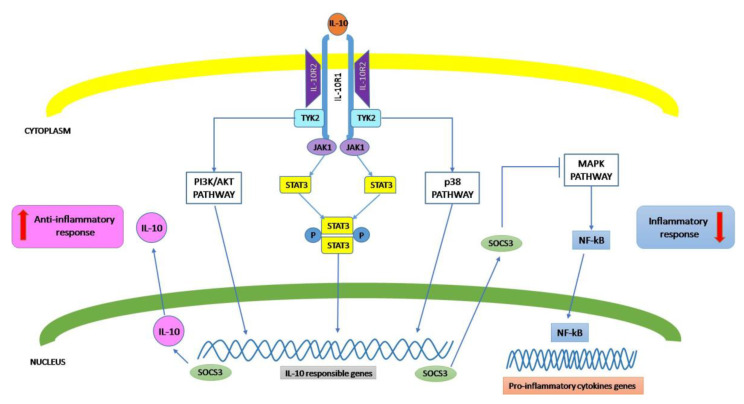
Scheme illustrating the IL-10 signaling pathway.

Upon binding to the cytokine, the receptor subunits engage molecules eliciting signal transduction in the cytoplasm of the cell that exhibits the functional receptor, leading to a signal that mainly influences the activity of the genes implicated in the immune response regulation. To date, the best characterized IL-10 signaling pathway is the Jak1/Tyk2/signal transducer and activator of transcription 3 (STAT3) system (Figure 1). IL-10 binding to its receptors triggers the recruitment of Jak1 to the IL-10Rα chain and its subsequent phosphorylation, while Tyk2 is recruited by the IL-10Rβ chain, and then, is phosphorylated [18,19]. These kinases, upon their phosphorylation, phosphorylate the tyrosine motifs Y446 and Y496 located in the intracellular portion of the IL-10Rα chain. In this context, it was reported that macrophages from Jak1−/− mice do not respond to IL-10, indicating the importance of Jak1 as an obligatory step in IL-10 signaling [20].

Phosphorylated STAT3 forms homodimers which translocate into the nucleus driving the expression of STAT3-responsive genes, that, among others, include the suppressor of cytokine signaling 3 (SOCS-3) [21]. Through SOCS-3 de novo synthesis, IL-10 has the capacity to inhibit the expression of a number of pro-inflammatory genes, including LPS-inducible cytokine genes, as TNF-α, IL-1, IL-6, IL-8, and IL-12; the IFN-γ inducible genes (MHC class II molecules, CD80/CD86, intercellular adhesion molecule-1 and inducible nitric oxide synthase); IL-4-inducible genes (type-I and type-II IL-1 receptors and CD23b). In summary, IL-10 exerts anti-inflammatory effects through inhibiting the production of pro-inflammatory cytokines, as well as preventing antigen presentation blocking major histocompatibility complex (MHC) class II expression and co-stimulatory molecules such as CD80/CD86 [22]. In addition, SOCS-3 inhibits mitogen-activated protein kinase (MAPK) activation, NF-κB translocation into the nucleus, and the associated induction of pro-inflammatory gene expression. Finally, SOCS-3 mediates Jak1-inhibition, resulting in feedback inhibition of the Jak1/Tyk2/STAT3 pathway [23].

However, other signaling pathways have been described for IL-10. In fact, previous research conducted on IL-10 receptor-signaling pathways reported that Jak1/STAT3-dependent signaling is not sufficient for the anti-inflammatory effect exerted by IL-10. In this regard, it was observed that IL-10 can promote the expression of heme oxygenase-1 (HO)-1, through the engagement of the p38-dependent pathway. HO-1, a stress-inducible protein with anti-inflammatory action, is the rate-limiting enzyme involved in the catabolism of heme, determining the generation of biliverdin, free iron and carbon monoxide (CO). This last one is able to neutralize the expression of induced pro-inflammatory cytokines, increasing, at the same time, IL-10 expression both in mouse primary macrophages and in the J774 cell line after LPS activation [22,24]. Interestingly, it was reported in LPS-activated macrophages that co-treatment of a HO-1 inhibitor or a CO scavenger decreases the inhibitory effects of IL-10 on TNF-α and NO production, as well as the expression of matrix metalloproteinase-9. Finally, HO-1 induction was also detected in mice receiving IL-10 administration. These observations enforce the idea that both HO-1 and CO play critical roles in mediating the anti-inflammatory effect of IL-10 both in vitro and in vivo [24,25].

Several studies support that the IL-10/STAT3 signaling pathway is involved in chronic stress-induced immune suppression. According to these reports, it was demonstrated that STAT3-deficient DCs exhibit an enhanced immune activity, such as increased cytokine production, Ag-dependent T cell activation other than resistance to IL-10-mediated suppression [26,27]. Williams et al. [27] reported that STAT3 is fundamental to mediate the IL-10 anti-inflammatory effects, proposing the existence in macrophages of a constitutively active form of STAT3 (STAT3C). In this case, STAT3C is able to mimic the inhibitory activity of IL-10, dampening LPS-induced TNFα and IL-6 production, thus, demonstrating that STAT3 is the only signal required to convey the major anti-inflammatory activity of IL-10 within human myeloid cells [27]. Furthermore, it was reported that STAT3 deletion increases, in the tumor microenvironment, cytotoxic activity exerted by natural killer cells other than increasing IL-12 production by DCs [28]. Finally, displacement of Th1/Th2 cytokine balance determined by chronic stress was also recovered by blocking IL-10/STAT3 axis [29].

## 4. IL-10 in the Brain

Glial cells in the CNS are basically engaged in maintenance of neuronal homeostasis, avoiding the onset and progression of potentially dangerous neuroinflammatory processes. In this regard, microglia, representing the resident macrophages, are, in the brain, the only CNS cells of hematopoietic origin, others being glial cells, as neurons, derived from neuroectoderm [30]. It has been well accepted that macrophages can modify their metabolic functions from a heal/growth promoting (M2 macrophages) to a killing/inhibitory phenotype (M1 macrophages) [31]. The diversity of microglia actions may be related to their ability to exhibit different activation stages—classical (M1) and alternative (M2), as described for tissue macrophages. The M1 phenotype is characterized by the production of pro-inflammatory mediators, including cytokines, such as TNFα, IL-6 and IL-1β; chemokines; reactive oxygen species; nitric oxide; prostaglandins. The neuroprotective M2 phenotype is characterized by the expression of IL-10, TGF-β and arginase and takes part in the resolution of inflammatory processes and tissue repair [32].

The generation of the anti-inflammatory cytokines as IL-10 constitutes one of the most sophisticated mechanisms implemented by immune cells to stem excessive inflammation and this aspect has paved the way for numerous studies focused to understand IL-10 regulation by cells present in CNS. It follows that IL-10 production is submitted to a fine regulation by a number of signal molecules, including glutamate, prostaglandin E2, mycoepoxydiene; all these compounds are able to enhance IL-10 release by microglia cells via TLR-4 activation [33,34]. In this regard, it has been reported that resveratrol treatment of microglia cells is able to counteract LPS-induced inflammatory effects through IL-10 upregulation [35].

Interestingly, IL-10 deficient mice show uncontrolled inflammation and increased susceptibility to bacterial, parasitic and viral infections in the CNS [36,37].

In the brain, IL-10 or IL-10R expression was described not only in microglia, but also in astrocytes, oligodendrocytes and neurons injury, as previously reported [38]. In addition, recent studies showed that IL-10Rα is expressed in retinal ganglion cells and in spinal cord neurons [39,40], suggesting that IL-10, probably, may modulate neuron functions independently from microglia and astrocytes. Therefore, the previous observations underlie the pivotal role for IL-10 in limiting neuroinflammatory processes in the brain, similarly to what happens in peripheral sites influencing resident macrophages in order to contain their inflammatory responses and promote the mechanisms ensuring tissue integrity.

## 5. IL-10 in Brain Diseases

Multiple sclerosis (MS) is an immune-mediated, inflammatory disease characterized by multifocal areas of demyelination in the CNS. The exact cause of MS is not yet well clarified, but it was reported that increased cytokine levels seem to play a crucial role in its pathogenesis, although it is not clear whether it leads to a beneficial or harmful effect. Among these cytokines, TNFα, IFN-γ, IL-1, IL-6, and IL-12 resulted positively correlated to the severity and progression of MS. Apart from the aforementioned cytokines involved in pro-inflammatory effects observed in the CNS of both MS patients and in animal models, there is evidence for the presence of several other cytokines, including IL-10, actively implicated in anti-inflammatory events in an attempt to assure the axonal and myelin integrity [41].

The genetic polymorphism related to the IL-10 gene responsible for the reduced expression of this cytokine has been related to the onset of MS symptoms in patients [42]. These observations were confirmed by what was found in mouse models of experimental autoimmune encephalomyelitis (EAE), in which the increase in IL-10 levels leads to symptoms reduction [43]. In this regard, other authors also reported that IL-10 expression in the CNS correlates to the onset of the recovery phase of EAE [44,45].

Although MS is primarily considered a T cell-mediated disease, successful B cell depletion with anti-CD20 monoclonal antibodies [46] suggests that these cells play an indispensable role in MS pathogenesis. Recently, it was also observed that B cells exhibit regulatory functions through TLR and CD40-mediated IL-10 production. In MS, TLR4- or TLR9-mediated signaling plays distinct roles in regulating IL-10 production by B lymphocytes. B cells from MS patients are deficient in their capacity to produce IL-10 after either TLR9 or CD40 stimulation, whereas TLR4-mediated IL-10 production was restored to normal levels in MS and further increased at relapse in the presence of CD40 signaling, thus, demonstrating that regulation of TLR4 and CD40 signaling in B cells may be a promising novel approach for MS therapy [47]. Consistent to this finding, previous studies reported that CD40 stimulation triggered continuous IL-10 production by human B cells in response to TLR stimulation correlating with B cell-mediated recovery from EAE by IL-10 production [48,49], promising a novel therapeutic approach for MS.

Furthermore, the employment of reinfusion of CD40-dependent IL-10-secreting autologous functional B cells was resulted to be an innovative and efficacious in vivo treatment for severe autoimmune diseases resistant to current therapies, including MS [50].

Traumatic brain injury (TBI) is characterized by a complex devastating injury with a broad spectrum of symptoms and disabilities, in which neuroinflammation plays a crucial role, being both protective and detrimental for brain compartments.

In this context, the release of mediators causing inflammation, such as pro-inflammatory cytokines and free radicals, although mainly produced for guaranteeing nervous tissue repair, is responsible for blood–brain–barrier (BBB) damage, leading to ischemia and cerebral edema [51]. It is well documented that IL-10 levels in humans and experimental animal models resulted significantly augmented both in the serum and CSF after TBI tending to remain elevated several days later [51]. Different experimental models, both in vitro and in vivo, evidenced that in ischemic strokes, IL-10 is implicated in neuroprotective action other than being an interesting and clinically useful diagnostic tool in TBI patients [52,53,54].

Conversely, other studies reported that elevated IL-10 levels correlate with severity and mortality in severe TBI [55]. In addition, higher levels of IL-10 in CSF were significantly associated with mortality both in pediatric and in adult patients [56].

A recent study reported that transplantation of mesenchymal stem cells engineered to overexpress IL-10 can reduce inflammation, determining a favorable outcome to the injured area. This result has been explained through the shifting of macrophages from pro-inflammatory to a pro-repair phenotype, thus, emphasizing the possible employment of IL-10 overexpression as a new therapeutic strategy for TBI treatment [57].

Among neurodegenerative diseases, Amyotrophic lateral sclerosis (ALS) is characterized by a rapid loss of motor neurons, both in the brain and spinal cord, that leads to paralysis and ultimately, death, in which neuroinflammation is one of the more frequently investigated key features [58]. Recent studies conducted in an animal model of ALS evidenced a specific role for IL-10 in the early stage of disease, clearly demonstrating that this cytokine plays a crucial role in orchestrating the immune responses of microglial cells [59]. In this regard, targeted overexpression of IL-10 in microglia seems to have therapeutic potential in ALS, since IL-10, assuring a microglial neuroprotective phenotype, delays neuronal dysfunction. As confirmation of this last observation, it is noteworthy that IL-10 production becomes deregulated with aging, resulting in reactive microgliosis and neuronal stress [60].

Other observations revealed characteristic changes in the levels not only of pro-inflammatory cytokines TNF-α and IL-6, but also of the anti-inflammatory cytokine IL-10, both in the spinal cord and in the serum of mice injected intraperitoneally with the IgG from the ALS patients accompanied with subclinical signs of motoneuron diseases, thus, evidencing the importance of the delicate balance between the pro- and anti-inflammatory mechanisms in neurodegeneration such as ALS [61]. Moreover, it was reported that IL-10 levels resulted high in ALS patients exhibiting a slowly progressive course or mild symptoms, thus, suggesting a possible neuroprotective action by this cytokine [62]. Su et al. [63] showed that depending on the cytokines found in plasma of ALS patients, shorter (IL-1β and IL-12) or longer (IL-10) disease duration can be predicted, thus, suggesting that a lesser degree of inflammation might be associated with more favorable prognosis. In another study described by Ehrhart [64], no significant differences were observed in terms of anti-inflammatory IL-4 and IL-10 cytokine concentrations in ALS patients versus control subjects. Therefore, at present, there is little information regarding the exact role of IL-10 during ALS in order to reach conclusive outcomes.

In a recent study, Jin M. et al. have studied the immune profile of peripheral blood and the serum cytokine pattern of 73 ASL patients, finding that the immune profile was shifted towards a Th1/Th17 cell-mediated pro-inflammatory response. Moreover, because the serum pro-inflammatory cytokines such as IL-1β, IL-6 and IFN-γ increased, whereas the anti-inflammatory IL-10 decreased, the authors correlated these events with disease severity and progression [65].

Alzheimer’s disease (AD), the most common cause of dementia in older adults, is a chronic neurodegenerative disease characterized by progressive loss of brain cells, formation of extracellular amyloid β (Aβ) plaques and intracellular neurofibrillary tangles [66]. Although the role of inflammation in AD is still uncertain, the increased expression of inflammatory mediators in the brains of AD patients and several epidemiological studies evidence a link between the use of anti-inflammatory drugs and the course of disease.

Pro- and anti-inflammatory cytokines are both decisive for the Aβ plaques’ onset in the brains of AD patients. In the aging brain, a combination of cytokines, such as IL-1β, IL-6, IL-8, IL-10, IL-12 and TNFα, is reported to trigger inflammatory processes associated with cortical atrophy [67].

To evaluate cerebral inflammation in AD, amnestic Mild Cognitive Impairment (aMCI) patients and aged matched healthy volunteers, Cisbani G et al. used radioligands targeting TSPO, a translocator protein strongly expressed in microglia and macrophages during inflammation, in conjunction with positron emission tomography (PET) imaging. Analyzing the association between candidate peripheral biomarkers (including amyloid beta, cytokines and serum total fatty acids) with brain TSPO levels, they found that serum IL-6 and IL-10 are higher in AD compared to the aMCI and healthy volunteers, whereas serum amyloid beta, cytokines and fatty acids were generally not correlated with neuroinflammation [68].

Moreover, apart from evidence reporting that IL-1 β is the most important pro-inflammatory cytokine contributing to an increased AD incidence, a weak expression of anti-inflammatory cytokines, including IL-10, exposes the subjects to a greater susceptibility to develop disease [63]. In this regard, IL-10 overexpression in the hippocampus of AD transgenic mice has been reported to increase neurogenesis and enhance cognition, thus, evidencing the probable neuroprotective role played by IL-10 in this pathological condition [69].

Conversely, other studies seem to support a detrimental effect by IL-10 causing Aβ clearance, inhibition in microglia, and worsening cognitive decline in AD mouse models [70]. IL-10 genetic ablation in APP/PS1 mice led to significant decline of the area interested by Aβ plaques’ presence, both in the cortex and hippocampus. In addition, it was observed that the severity of cerebral amyloid angiopathy, characterized by the deposition of Aβ within the blood vessels walls, was also reduced in this animal model [71]. On the other hand, a recent study [72] reported that promoter haplotypes of IL-10 may be important modulators of the development of amnestic mild cognitive impairment, thus, confirming previous observations regarding the linkage of polymorphisms in the promoter region of IL-10 and risk factors of AD [73].

Parkinson’s disease (PD) is a progressive nervous system disorder due to nigrostriatal dopamine neurons loss and is characterized by several clinical features, including bradykinesia, rigidity, tremor, and postural instability. Neuroinflammation is an important risk factor that may contribute to PD pathogenesis, since PD patients suffer from chronic inflammation that probably precedes neurodegeneration and cytokines produced by activated microglia in the substantia nigra (SN) and putamen in the course of PD [74].

It was observed in the brains in the LPS-induced PD mouse model that IL-10 decreased the number of activated microglia with a protective effect regarding the loss of dopaminergic neuron [75]. Moreover, PD patients with more severe clinical signs and a prognostically unfavorable non-tremor form show drastically reduced serum levels of IL-10 [76]. Alternatively, high plasma IL-10 levels have been detected in PD patients with classical motor symptoms in comparison to healthy controls [77]. However, in another study, it was reported that IL-10 levels seem not to be correlated to non-motor symptoms [78]. In addition, Li et al. detected both elevated levels of IL-1β and depressed levels of IL-10 in the peripheral blood of patients with PD-related pain, thus, suggesting the implication of several inflammatory cytokines, including IL-10, in the occurrence of PD-related pain [79].

Various studies have investigated a possible correlation between IL-10 polymorphisms and potential risk of PD onset. In this regard, some observations evidenced no association [80,81], although other studies showed that IL-10 promoter polymorphisms−819 and −1082 seem to be associated with PD risk and early PD occurrence [82].

A recent report showed increased peripheral concentrations of IL-6, IL-1β, TNFα, IL-2 and IL-10 in patients with PD [83]. Similarly, in the analyses of newly diagnosed PD patients, IL-1β, TNFα, IL-2 and IL-10 resulted elevated [84]. Although IL-10 generally has effects able to oppose the actions of the pro-inflammatory cytokines, its bioactivity is highly complex in the immunoregulation, including both immunosuppressive as well as immunostimulatory activities, as previously reported [1].

Interestingly, a correlation between IL-10 levels and gastrointestinal symptoms in the early stage of PD was also recently reported, thus, reflecting a protective response against inflammatory processes associated with the disease [85].

These observations suggest not only that certain inflammatory cytokines may be implicated in the occurrence and clinical symptoms of PD, but also that IL-10 may constitute a potential target for the development of new drugs.

## 6. Therapeutic Potential of IL-10-Based Therapies in Neurodegenerative Diseases

Most of the supporting studies for IL-10-based therapies in neurodegenerative diseases have been conducted in animals.

Richwine et al., reported that in IL-10-deficient mice, LPS peripheral administration causes a noticeable cognitive deficit unlike what can be observed in wild-type mice [86]. The concept that IL-10 may counteract neurodegeneration was also supported by observations in transgenic AD mouse models, in which IL-10 is able to significantly reduce neuroinflammation, enhancing neurogenesis and improving spatial cognitive dysfunction [71]. These findings are supported by studies evidencing that post-menopausal administration of estrogens is responsible for the increase in IL-10 release by microglia, that, in turn, contributes to AD prevention [87,88]. Moreover, treatment with resveratrol, a natural polyphenol with anti-inflammatory effects, is described to upregulate both IL-10 gene expression and IL-10 levels, with neuroprotective actions [35]. Confirming this result, Bagyinszky et al., reported that IL-10 treatment could represent a potential therapy for AD, since this anti-inflammatory cytokine downregulating the pro-inflammatory cytokine expression could determine amyloid reduction [89].

In contrast, it was reported that in IL-10 overexpressing AD animal models, the defective phagocytosis of soluble Aβ by microglia exacerbated Aβ deposits causing cognitive impairment. A possible explanation of this effect may be that IL-10 driving macrophage polarization from M1 to M2 could lead to microglia deactivation, increasing Aβ accumulation and worsening behavioral deficits. Supporting this concept are studies demonstrating that knockout mice showed the benefit of IL-10 removal, resulting in decreased cognitive and synaptic dysfunctions and promoting Aβ clearance [73].

Interestingly, a recent work reported as possible drug candidate sitagliptin, producing, among its biological effects, an IL-10 increase in the treatment of certain neurodegenerative disorders linked with neuroinflammation, with special emphasis on AD [90].

A recent work reported that IL-10 pre-treatment may protect primary rat ventral mesencephalic neurons against toxic actions of LPS through the inhibition of TNFα release and upregulating Brain-derived neurotrophic factor (BDNF).

Levels. At the same time, IL-10 suppressed the upregulation of active forms of caspase-3 and caspase-9 induced by LPS reducing neuronal apoptosis, thus, suggesting that IL-10 has neuroprotective effects against LPS by downregulating pro-inflammatory cytokines mediated neuronal apoptosis [91].

Confirming these observations, it was reported that loss of dopaminergic neurons, due to the intra-SN injection of LPS in mice, resulted selectively reduced after SN osmotic infusion of IL-10, containing microglial activation [92].

In the in vitro cerebral ischemic model, built with cortical neurons exposed to oxygen-glucose deprivation (OGD), IL-10 produced a dual effect on the apoptosis at different OGD stages through the p65 pathway at an early stage and c-Rel pathway at a late stage, and it seems that IL-10 administration showed an optimal neuronal protective effect at a middle-to-late stage after OGD. These findings demonstrated the potential therapeutic value of IL-10 in cerebral ischemia [93].

Moreover, in a 1-methyl-4-phenyl-1,2,3,6-tetrahydropyridine (MPTP) model of PD, which causes striatal tyrosine hydroxylase depletion and subsequent dopaminergic cell death, the adeno-associated viral type-2 vector containing the complementary DNA for human IL-10, injected intracerebroventricularly before MPTP injection, provided neuroprotective effects by increasing striatal tyrosine hydroxylase [94].

Furthermore, in the same animal PD model, it was reported that vitamin D, apart from mitigating pro-inflammatory cytokines expression in different brain areas, can upregulate IL-10, CD163, CD206 and CD204 expression, typical hallmarks of M2 microglia alternative activation. In this regard, the neuroprotective action done by vitamin D was attributed to upregulation of IL-10, in turn, shifting the microglial population from a pro-inflammatory phenotype to an anti-inflammatory one [95].

In this regard, in another PD animal model represented by a 6-OHDA rat model, it was reported that Fluvoxamine treatment attenuated neuronal inflammation, reducing lipid peroxidation, as well as IL-1β, IL-6 and TNFα mRNA levels, while IL-10 and TGF-β levels resulted upregulated in the striatum of treated animals [96].

Recently, vinpocetine was reported to be a good candidate for possible PD therapy. In fact, in vivo administration of vinpocetine reduced serum levels of inflammatory cytokines, TNFα and MCP-1, increasing significantly IL-10 [97].

Immunotherapy and gene therapy are reported to play promising roles in modern medicine for the treatment of neurodegenerative diseases. Synergistic expression of three cytokines, including IL-10, in Wharton’s jelly stem cells resulted in an effective cell and gene therapy in the EAE mice model, thus, promising a therapeutic approach against MS [98]. A genetic vaccine containing the hsp65 mycobacterial gene resulted able to reduce EAE clinical signs and trigger higher IL-10 production in the CNS, thus, evidencing how IL-10 may represent one of the most effective anti-inflammatory cytokines for MS control [99].

The alkaloid natural compound tetramethylpyrazine was reported, in EAE mice, to suppress the expression of pro-inflammatory cytokines IL-18 and IL-17 and trigger the expression of IL-10, thus, resulting in improvements of clinical manifestations by decreasing demyelination. This result emphasizes the use of a therapeutic agent able to limit neuroinflammatory response through the IL-10 upregulation for MS treatment [100].

Other studies conducted in animal models described beneficial effects of IL-10 administration both in brain ischemia [101] and in PD [102]. However, in some situations, such as in AD, a detrimental role for IL-10 in the brains of APP transgenic mouse models in terms of increased Aβ accumulation and impaired memory was described [71,103]. In any case, it clearly appears that modulating IL-10 production in the brain may represent an attractive strategy to recompose, unlike detrimental immune responses. In this perspective, the in-depth knowledge of the molecular mechanisms regulating IL-10 production in the brain, still often poorly understood, constitutes a research field that deserves attention.

## 7. Conclusions

Considering the previous observations, it seems that IL-10 must be correctly modulated in the context of neurodegenerative diseases, so that its production does not result in excessive quantities to prevent the elimination of potentially harmful agents and, at the same time, its levels are able to effectively balance the pro-inflammatory responses.

**Figure 2:**
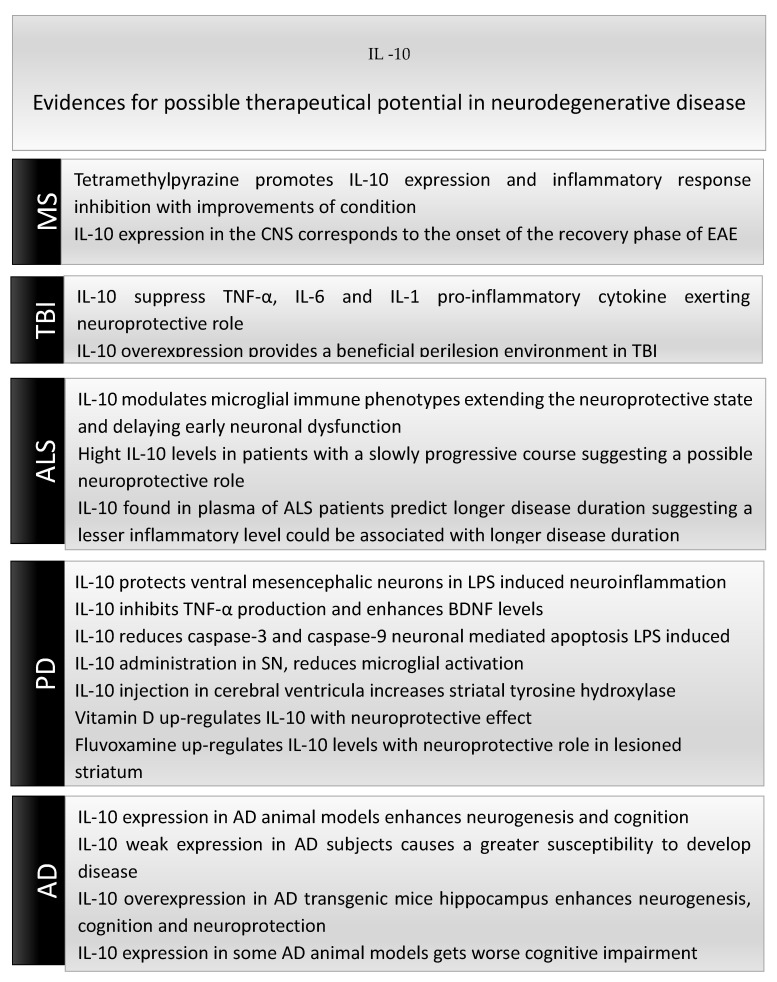
Potential therapeutic actions of IL-10 in neurodegenerative diseases.

Figure 2 summarizes the potential therapeutic effects of IL-10 in neurodegenerative diseases.

IL-10 administration seems to have a beneficial effect in most of the neurodegenerative diseases analyzed in this narrative review. Considering the potential of IL-10 in brain immune responses’ orchestration, it is of fundamental importance to understand the molecular mechanisms that regulate IL-10 expression in neuroinflammatory processes in view of future therapeutic approaches.
